# Habitat structure alters top-down control in litter communities

**DOI:** 10.1007/s00442-012-2530-6

**Published:** 2012-11-28

**Authors:** Gregor Kalinkat, Ulrich Brose, Björn Christian Rall

**Affiliations:** 1 J. F. Blumenbach Institute of Zoology and Anthropology, Georg-August-University Göttingen, Berliner Str. 28, 37073 Göttingen, Germany; 2 Department of Biology, Technische Universität Darmstadt, Schnittspahnstr. 10, 64287 Darmstadt, Germany

**Keywords:** Bottom-up control, Functional response, Non-linear interaction strength, Predator–prey interaction, Soil food webs

## Abstract

The question whether top-down or bottom-up forces dominate trophic relationships, energy flow, and abundances within food webs has fuelled much ecological research with particular focus on soil litter ecosystems. Because litter simultaneously provides habitat structure and a basal resource, disentangling direct trophic and indirect non-trophic effects on different trophic levels remains challenging. Here, we focussed on short-term per capita interaction strengths of generalist predators (centipedes) on their microbi-detritivore prey (springtails) and addressed how the habitat structuring effects of the leaf litter modifies this interaction. We performed a series of laboratory functional response experiments where four levels of habitat structure were constructed by adding different amounts of leaf litter to the experimental arenas. We found that increased leaf litter reduced the consumption rate of the predator. We interpreted this as a dilution effect of the augmented habitat size provided by the increasing leaf litter surface available to the species. Dilution of the prey population decreased encounter rates, whereas the capture success was not affected. Interestingly, our results imply that top-down control by centipedes decreased with increasing resource supply for the microbi-detritivore prey (i.e. the leaf litter that simultaneously provides habitat structure). Therefore, effective top-down control of predators on microbi-detritvore populations seems unlikely in litter-rich ecosystems due to the non-trophic, habitat-structuring effect of the basal litter resource.

**Electronic supplementary material:**

The online version of this article (doi:10.1007/s00442-012-2530-6) contains supplementary material, which is available to authorized users.

## Introduction

Progress in food-web ecology is critically based upon information about bioenergetic flows of energy between consumer and resource pairs. These interaction strengths and their distributions across the myriads of links in natural food webs are vital for community structure, population dynamics, and ecosystem functioning (e.g. McCann et al. 1998; Neutel et al. 2002, 2007; Otto et al. 2007; Rall et al. 2008; Berlow et al. 2009; Binzer et al. 2011). The biotic mechanisms shaping and structuring interaction strengths are complex and might be driven by basal resources (e.g. detritus) or consumers (e.g. predators). One major question in the ecology of soil food webs therefore deals with the regulation of detritivore populations and whether they are controlled by bottom-up mechanisms (i.e. energy and nutrient supply) or top-down regulated by their multiple predators. Both hypotheses are supported by studies: Bengtsson et al. (1997) found top-down control, whereas the results of Scheu and Schaefer (1998) and Ponsard et al. (2000) provided evidence for bottom-up control. Major progress in this field requires insights in consumer–resource interactions with a particular focus on the strength of such interactions (Scheu 2002). Due to the natural composition of soil and litter habitats with their porous, fractal structure and opaqueness, the direct observation of species interactions in the natural context is almost intractable. Indirect observation via gut or stomach content analysis, a standard procedure in freshwater (e.g. Elliott and Persson 1978; Woodward and Hildrew 2002) and marine (e.g. Daan 1973; Aljetlawi et al. 2004; Smout and Lindstrøm 2007) systems is hampered by the fact that a large fraction of soil predators rely on extra-intestinal digestion (Cohen 1995) and therefore deep understanding of predator–prey interaction strengths in these systems remains challenging. While different methods of tracking feeding links *qualitatively* were developed and improved over the past decades—particularly stable isotope analyses, molecular gut content analyses and fatty acid trophic markers (Post 2002; King et al. 2008; Ruess and Chamberlain 2010)—they have scant ability for tracking feeding interactions *quantitatively*. Therefore, we have to rely on laboratory experiments to determine per capita impacts of litter- and soil-dwelling predators on their prey.

One well-established model framework for analysing interaction strengths is the functional response (Holling 1959; Berlow et al. 2004). It describes the density-dependent per capita consumption rate, *F*_*ij*_, of a predator *j* on a prey *i* (Holling 1959; Real 1977):

1 $$ {F_{ij} = \frac{{a_{ij} N_{i}^{q + 1} }}{{1 + a_{ij} h_{ij} N_{i}^{q + 1} }}} $$

where *F*_*ij*_ (n_*i*_ n_*j*_^−1^ day^−1^) is the per capita consumption rate (also referred to as intake rate, ingestion rate, predation rate or feeding rate), *N*_*i*_ [(n_*i*_ m^−2^) or (n_*i*_ m^−3^)] is prey density, *h*_*ij*_ (n_*j*_ day n_*i*_^−1^) is the handling time needed to kill, ingest and digest a resource individual, *a*_*ij*_ is the capture rate [(m^2^ day^− 1^ n_*j*_^−1^) or (m^2^ day^− 1^ n_*j*_^−1^)] and *q* is a scaling exponent converting the hyperbolic type II functional response (*q* = 0) to a sigmoid type III functional resonse (*q* = 1; Real 1977; Hassell 1978; Rall et al. 2008; Vucic-Pestic et al. 2010b). Note that the capture rate (often also referred to as “attack rate” or more accurately “rate of successful attacks”) is expressed on a movement or velocity scale [with either area or volume depending on the foraging mode of the predator with each prey and the ecosystem type where predator and prey occur (McGill and Mittelbach 2006; Rall et al. 2012; Pawar et al. 2012)]. It includes the rates of encounter and success of attacks (Gergs and Ratte 2009; Vucic-Pestic et al. 2011).

Generally, two different approaches to determine capture rates and handling times can be distinguished: (1) direct observation and (2) indirect derivation through model fitting. In carefully designed experiments, both approaches result in congruent parameter estimates (Tully et al. 2005). While direct observation is feasible, particularly for larger predators (e.g. fishes in laboratory experiments; Persson and Brönmark 2002) studies working with diminutive organisms in opaque environments have to either rely on adequate model fitting techniques to reveal functional response parameters or reduce the structural complexity of the experiment to improve the visibility of the interactions. As Jeschke et al. (2004) highlighted, the majority of functional response studies are carried out in simplified laboratory systems. The resulting problem that feeding rates might differ in more complex experiments has been addressed by several functional response studies in recent years: there, experimental complexity was introduced by variation of numbers of predator individuals (predator interference; e.g. Kratina et al. 2009; Lang et al. 2012), the number of prey species (alternative prey; e.g. Colton 1987; Elliott 2004; Kalinkat et al. 2011) or even the additional presence of non-prey species (Kratina et al. 2007). Another lack of reality in laboratory studies is due to oversimplified environmental conditions that are typically provided within artificial arenas. There are only a limited number of studies focussing on the effects of habitat complexity on the functional response of terrestrial predators (Kaiser 1983; Munyaneza and Obrycki 1997; Pitt and Ritchie 2002; Hoddle 2003; Hohberg and Traunspurger 2005; Hauzy et al. 2010; Vucic-Pestic et al. 2010a). While some of these studies focussed on the fractal complexity of an artificially structured habitat (Kaiser 1983; Pitt and Ritchie 2002; Hoddle 2003) and others made qualitative comparisons of with-structure- versus non-structure-treatments (Hohberg and Traunspurger 2005; Vucic-Pestic et al. 2010a), there is only one study to our knowledge with a qualitative comparison between a simplified, unstructured laboratory setting and field conditions (Munyaneza and Obrycki 1997). This study indicated reduced capture rates of terrestrial arthropod predators by a factor of roughly two under greenhouse and field conditions compared to the experimental setting with controlled conditions in the laboratory experiment.

Beyond these specific functional response studies, a broader look at the literature reveals that habitat structure effects on predator–prey interactions have been the focus of many studies especially in aquatic ecosystems (e.g. Crowder and Cooper 1982; Gotceitas and Colgan 1989 and references therein). There, predation rates are also reduced in high-complexity treatments and tend to be highest in habitats with intermediate structural complexity (Crowder and Cooper 1982). However, a continuous framework that is suitable to link trophic and non-trophic effects between basal resources (e.g. litter), first-order consumers (e.g. detritivores) and predators is still missing. This applies particularly to leaf litter systems, where pulses of incoming material and long-lasting decay of the litter yield a continuously changing amount and complexity of habitat structure. Therefore, our understanding of dynamics and functioning of such ecosystems is challenged by a general lack of studies addressing how habitat structure modifies interaction strengths and top-down control of microbi-detritivores by predators.

In this study, we aimed to fill this gap by studying the effects of systematic variation in leaf litter density on the functional response of the centipede *Lithobius mutabilis* (Chilopoda: Lithobiidae) as a ubiquitous and frequent generalist predator of the leaf-litter system on its microbi-detritivore prey, the springtail *Heteromurus nitidus* (Collembola: Entomobryidae). Springtails have been shown to be flexible foragers that can feed on fungal hyphae, bacteria or detritus depending on the available resources (Scheu 2002). According to Lawrence and Wise (2000), they might be assigned to the functional guild of detritivores as higher abundances of springtails co-occurred with increased rates of litter disappearance in this experiment. Within the model framework of the functional response, we expected prey refuges of the additional habitat structure to cause a shift from type II to type III functional responses (Real 1977; Scheffer and De Boer 1995), as has already been shown for other predator–prey pairs from litter systems (de Ruiter et al. 1988, Vucic-Pestic et al. 2010b). Furthermore, we anticipated that the capture rate should be negatively affected by increasing habitat structure as encounter rates are directly dependent on movement patterns and velocities of predators and prey (Muirhead and Sprules 2003; Gergs and Ratte 2009). Therefore, habitat complexity should only affect the encounter rate and not the mechanisms involved once the two species are in close contact (which includes handling time).

In consequence, we hypothesised that the increased complexity of leaf litter should (1) provide additional prey refuges therefore resulting in more sigmoid type III functional responses, (2) decrease the capture rates, and (3) not affect the handling times.

## Materials and methods

### Functional response experiments

The basic experimental set-up follows prior functional-response experiments (Vucic-Pestic et al. 2010b, 2011; Rall et al. 2011). We studied the per capita consumption rates of the centipede *L. mutabilis* on the springtail *H. nitidus* at varying prey densities from 1 to 1,000 individuals of springtails per arena (corresponding to 25–25,000 individuals per m^2^) at four levels of habitat complexity (1, 2, 4 and 8 g dry weight of beech litter corresponding to 25, 50, 100 and 200 g/m^2^, respectively). Each prey density was replicated three to five times resulting in a total number of 123 experimental units. The centipedes were sampled by hand from field sites in the Hainich-Dün National Park, Thuringia, Germany. Freshly fallen beech litter was sampled at the same sites. The predator individuals were kept separate from each other in moistened plastic jars and were deprived of food for at least 48 h before the start of the experiments. The experiments were performed in Perspex^®^ arenas (0.2 × 0.2 × 0.1 m) covered with lids with holes to allow gas exchange. The arena floor was covered with moist plaster of Paris (200 g dry weight) to provide constant moisture during the experiments. Beech litter for providing habitat structure in the arenas was first dried for several days at 40 °C to eliminate other animals and then re–moisturised prior to the experiments. Prey individuals were placed in the arenas 30 min prior to the predators to allow them to adjust to the arenas. The experiments were run for 24 h with a day/night rhythm of 12/12 h dark/light and a constant temperature of 15 °C in temperature cabinets. Initial and final prey densities were used to calculate the number of prey eaten. Control experiments without predators showed that effects of prey mortality or escape were negligible. As recent studies have shown strong allometric effects on the functional responses of terrestrial invertebrate predators (Vucic-Pestic et al. 2010b; Rall et al. 2011, 2012), we controlled predator and prey weight and kept it at a constant level (centipedes: 22.74 ± 0.77 mg standard error; springtails: 0.15 ± 0.004 mg standard error). Note that, in this experimental design, we intentionally excluded trophic effects between the litter and the springtails. Short-term experiments with freshly fallen leaves that were not yet colonised by fungi or bacteria serving as potential resources for the springtails assured that we would reveal particularly the non-trophic effect of the leaf litter as habitat structure.

### Leaf area

Generally, leaf litter density is positively correlated with surface area available for the predator–prey interaction. Hence, expressing consumption and capture rates relative to the surface area of the experimental arenas might become arbitrary with increasing leaf litter density. In order to provide an alternative approach accounting for increases in surface area with increasing leaf litter density, we “corrected” the prey densities relative to the leaf surface area plus the arena area to get the “total foraging area”: Therefore, we measured the leaf surface area of a representative set of 12 samples of leaves (three replicates of 1, 2 and 4 g dry weight, respectively) that were used within the experiments. For each sample, we determined leaf surface area by optical scanning with a flatbed graphics scanner and subsequent analyses of the images with the software WinFOLIA, v.5.1a (Regent Instruments Inc., Quebec City, Canada). We fitted leaf area against leaf litter dry weight using a linear model. Subsequently, three different spatial scenarios were compared in our functional response analyses: (1) uncorrected area, i.e. 0.04 m^2^ arena surface area in all leaf litter treatments, (2) one-side corrected (hereafter, one-sided) area with the one-sided leaf area plus arena surface area, and (3) two-side corrected (hereafter, two-sided) area with the two-sided leaf area plus arena surface area (see Table 1 for an example how prey densities were corrected for differing habitat size according to these three scenarios).

**Table 1:** Results of leaf area linear model fit (with 95 % CI) and examples of deduced density correction factors for one individual per experimental arena (0.04 m^2^)

Leaf litter weight (g)	One-sided leaf area (m^2^) (±95 % CI)	Uncorrected (Ind/m^2^)	One-sided (Ind/m²)	Two-sided (Ind/m^2^)
1	0.0234 (±0.0020)	25	15.7633	11.5105
2	0.0469 (±0.0040)	25	11.5105	7.4764
4	0.0938 (±0.0080)	25	7.4764	4.3954
8	0.1875 (±0.0161)	25	4.3954	2.4095

### Statistical analyses

Initially, we fitted a polynomial logistic regression to the proportion of prey eaten to investigate the shape of the functional response (Juliano 2001):

2 $$ {\frac{{N_{e} }}{{N_{0} }} = p = \frac{{{\text{e}}^{{p_{0} \left[ L \right] + p_{1} \left[ L \right]N_{0} + p_{2} \left[ L \right]N_{0}^{2} + p_{3} \left[ L \right]N_{0}^{3} }} }}{{1 + {\text{e}}^{{p_{0} \left[ L \right] + p_{1} \left[ L \right]N_{0} + p_{2} \left[ L \right]N_{0}^{2} + p_{3} \left[ L \right]N_{0}^{3} }} }}} $$

where *p* represents the predation risk of one prey item to be killed, *N*_0_ is the initial prey density, *N*_*e*_ is the number of prey killed during the experiment, *p*_0_, *p*_1_, *p*_2_ and *p*_3_ are statistically estimated parameters and *L* represents the level of leaf litter density. In this vein, a continuously decreasing relationship of predation risk dependent on prey density indicates a type II functional response, whereas a hump-shaped curve indicates a type III functional response (de Ruiter et al. 1988; but see Juliano (2001) for detailed methodology). The goal was to identify possible differences in the shape of the responses between the different leaf litter density treatments. Therefore, we performed a stepwise backwards selection by firstly deleting the factorial litter density treatment levels, and afterwards the polynomial terms. Accordingly, we simplified the model until the Akaike Information Criterion (AIC) (Akaike 1974) indicated the best fit.

Subsequently, we used type II models in all following analyses, because decreasing functions for predation risk were identified as best models for all leaf-litter treatments (see “Results”). To avoid violation of our statistical results due to prey depletion during the course of the experiment, we then used the integrated form of the functional response, also known as Rogers ‘Random Predator Equation’ (Royama 1971; Rogers 1972) because Eq. (1) assumes a constant prey density throughout the experiment:

3 $$ {N_{e} = N_{0} \left( {1 - {\text{e}}^{{\left( {a_{ij} \left( {N_{e} h_{ij} - PT} \right)} \right)}} } \right)} $$

where *N*_*e*_ (n_*i*_ m^−2^) is the density of prey *i* eaten during the experiment, *P* is predator *j*’s density, *T* is the experimental time (days) and all other parameters are as in Eq. (1). We solved this recursive function of *N*_*e*_ with a non-linear least squares method (“nls”) using the additional package “emdbook” for the statistical software package R (Bolker 2008; R Development Core Team 2010). The resulting equation is

4 $$ {N_{e} = N_{0} - \frac{{W\left( {a_{ij} h_{ij} e^{{\left( { - \left( {PT - h_{ij} N_{0} } \right)} \right)}} } \right)}}{{a_{ij} h_{ij} }}} $$

where *W* is the Lambert W function (see Bolker 2008 and references therein for a detailed description). Furthermore, we analysed the effect of litter density on capture rates and handling times by inserting either exponential

5a $$ {a_{ij} = a_{0} {\text{e}}^{{\varepsilon_{a} L}} } $$

5b $$ {h_{ij} = h_{0} {\text{e}}^{{\varepsilon_{h} L}} } $$

or power law functions

6a $$ {a_{ij} = a_{0} L^{{\left( {b_{a} } \right)}} } $$

6b $$ {h_{ij} = h_{0} L^{{\left( {b_{h} } \right)}} } $$

in Eq. (4), where *a*_*0*_ and *h*_*0*_ are constants, *L* is the leaf-litter density, *ε*_*a*_ and *ε*_*h*_ determine the exponential increase or decrease of capture rates and handling times in dependence on leaf-litter density, while *b*_*a*_ and *b*_*h*_ are the scaling exponents of the power law functions. Additionally, functional response models with constant values of *a*_*0*_ and *h*_*0*_ without leaf litter dependence were also fitted to the data. We fitted all possible combinations of the three capture-rate models and three handling-time models (constant, exponential, power law) under each of the three spatial scenarios (uncorrected, one-sided and two-sided) to the data resulting in a total of 27 functional response models and ranked them according to their ΔAIC (see supplementary Table S1 for an overview).

## Results

**Fig. 1:**
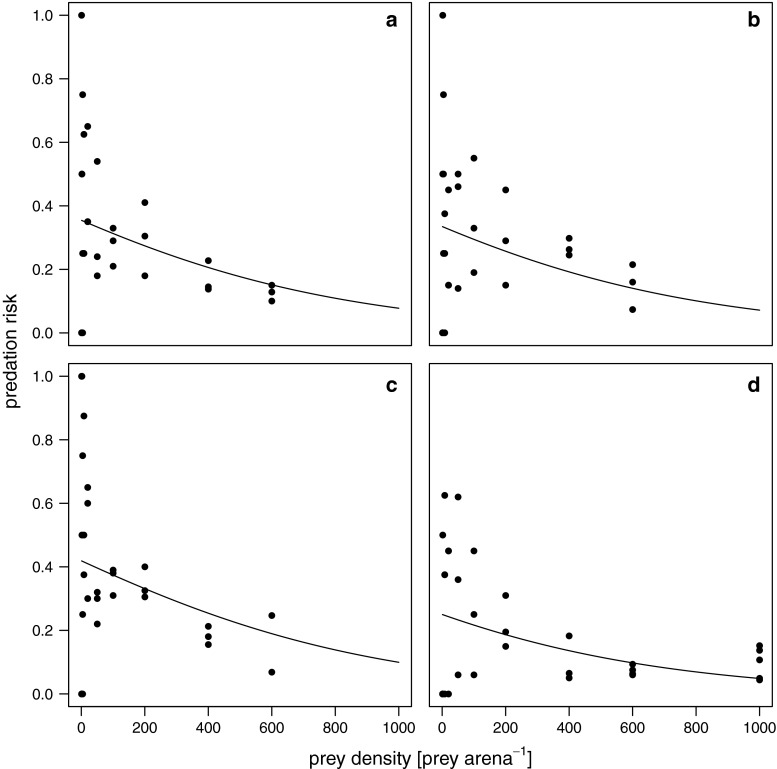
Results of the best-fitting polynomial regression model to test for the shape of the functional response depicting the different leaf litter density treatments of **a** 1 g, **b** 2 g, **c** four g and **d** 8 g leaf litter per arena, respectively. The model included the negative linear term *p*_*1*_ = −0.0019 (SE = 0.0007, *t* = −2.731, *p* < 0.01) and four litter-dependent constants: *p*_*0*_(1 g) = −0.5989 (SE = 0.2057, *t* = −2.911, *p* < 0.01), *p*_*0*_(2 g) = −0.6860 (SE = 0.2115, *t* = −3.243, *p* < 0.01), *p*_*0*_(4 g) = −0.3267 (SE = 0.1927, *t* = −1.696, *p* = 0.092) and *p*_*0*_(8 g) = −1.0978 (SE = 0.2639, *t* = −4.161, *p* < 0.001)

The logistic regression analyses showed that the best model only included the constant *p*_0_ in dependence of the leaf litter level and a negative linear term *p*_1_ (Fig. 1, see figure legend for statistical outputs). This result suggested a type II functional response that did not differ in shape for all four leaf litter levels whereas overall predation risks differed according to leaf litter density. Therefore, all subsequent analyses where made with a type II functional response model.

**Fig. 2:**
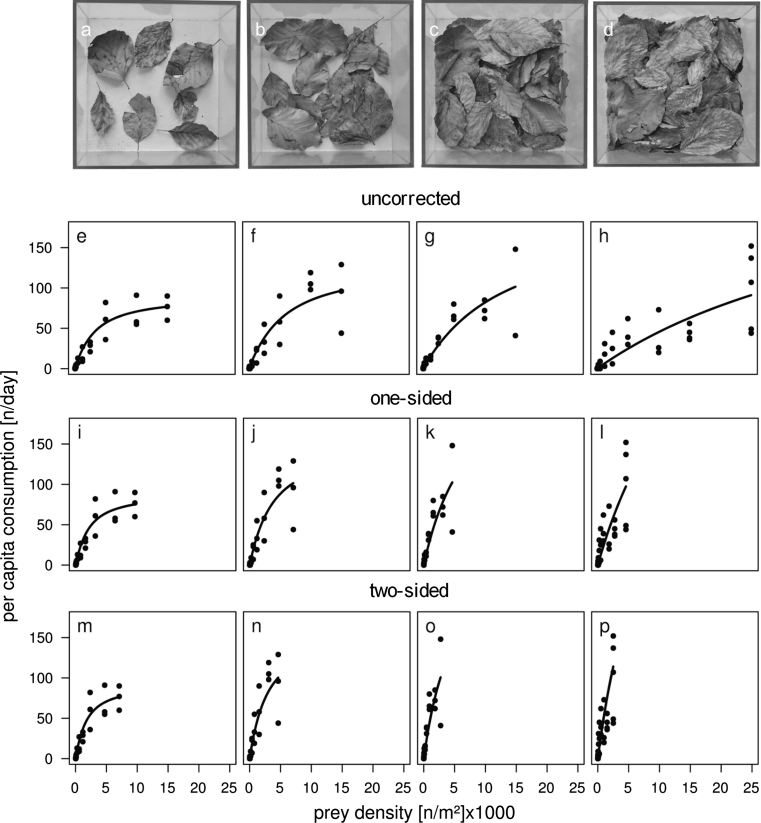
The leaf litter within the experimental arenas (0.04 m^2^ ground area) of the four treatments with **a** 1 g dry weight leaf litter, **b** 2 g, **c** 4 g and **d** 8 g. Beneath are the functional response curves according to the respective best-fitting model for the uncorrected (**e–h**), one-sided (**i–l**) and two-sided (**m–p**) densities. Parameter values are given in Table 2

The mean leaf area (one-sided) increased from 0.023 m^2^ (±0.002 95 % CI) in the treatment with 1 gram leaf litter to 0.188 m^2^ (±0.016 95 % CI) in the treatment with 8 g leaf litter (Table 1; Fig. 2a–d) following a linear model fit through the leaf areas of 1, 2 and 4 g dry weight of leaf litter (*n* = 12, *R* ^2^ = 0.984, *p* < 0.0001). This increase in the surface area available for animal movement and interactions implied that the prey density (here, for example, for one springtail individual per arena) decreased from 25 ind/m^2^ (uncorrected) to ~16 ind/m^2^ (one-sided) and ~12 ind/m^2^ (two-sided) in the treatment with 1 g leaf litter or to ~4 ind/m^2^ (one-sided) and ~2 ind/m^2^ (two-sided) in the treatment with 8 g leaf litter (Table 1; Fig. 2a–d). While prey densities were the same across treatments in the scenario with uncorrected area (Fig. 2e–h, second row), the increases in leaf surface area with the amount of leaf litter resulted in a shift in prey densities from higher densities in treatments with 1 g leaf litter (Fig. 2, left column) to lower densities (Fig. 2i–l: one-sided; m–p: two-sided).

**Fig. 3:**
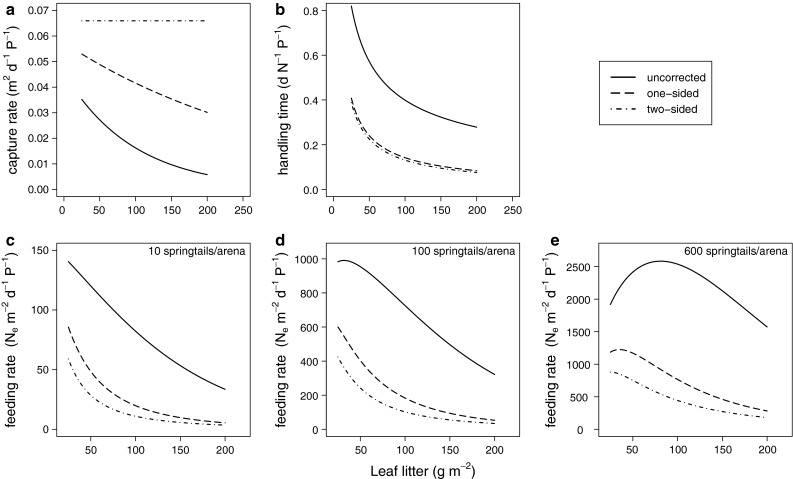
Relationship between leaf litter density and **a** capture rates and **b** handling times. Curves are based on the best-fitting functional-response models with uncorrected (*solid line*), one–sided (*dashed line*) and two-sided (*dash-dotted line*) prey densities. **c**–**e** show the resulting relationships for leaf litter density and consumption rates at 10 (**c**), 100 (**d**) and 600 (**e**) prey individuals per experimental arena

Capture rates showed decreasing exponential functions with increasing leaf litter density for the uncorrected and the one-sided prey densities, whereas functional-response models with a constant capture rate provided the best fit to the data under two-sided correction (Table 2; Fig. 3a). Comparing the parameter values of the models with exponential relationship for capture rates, there is a clear trend from a highly significant negative relationship for the uncorrected densities (ε_a_ = −0.0103, SE = 0.0014, *p* < 0.0001), a shallower slope with lower significance for the one-sided correction (ε_a_ = −0.0032, SE = 0.0014, *p* = 0.020) to a non-significant (constant) relationship under the two-sided correction (ε_a_ = −0.0016, SE = 0.0014, *p* = 0.267 in the second best model fitting; see Table 2 for parameter estimates for the best fitting models, respectively). Surprisingly, in all three spatial scenarios (uncorrected, one-sided and two-sided densities), the best-fitting model with the lowest ΔAIC included power law decreases in handling times with increasing leaf litter density (Tables 2 and S1; Fig. 3b). This contradicted our third initial hypothesis that handling time should not be affected by litter density. All the functional response models with constant handling time yielded a much poorer fit to the data (Table S1) suggesting that our third hypothesis had to be rejected.

**Table 2:** Parameter estimates for best model fittings for uncorrected, one-sided and two-sided densities, respectively

	Parameter estimate	SE	*t* value	*p*
Uncorrected				
*a*_0_	0.0456	0.0118	3.877	<0.001∗∗∗
*ε*_a_	−0.0103	0.0014	−7.364	<0.0001∗∗∗
*h*_0_	0.0586	0.0354	1.653	0.101
*b*_h_	−0.5196	0.1675	−3.102	0.002∗∗
One-sided				
*a*_0_	0.0574	0.0109	5.289	<0.0001∗∗∗
*ε*_a_	−0.0032	0.0014	−2.361	0.020∗
*h*_0_	0.1297	0.0730	1.777	0.078∙
*b*_h_	−0.7618	0.1694	−4.497	<0.0001∗∗∗
Two-sided				
*a*_0_	0.0659	0.0086	7.705	<0.0001∗∗∗
*h*_0_	0.1340	0.0777	1.723	0.088∙
*b*_h_	−0.7839	0.1810	−4.331	<0.0001∗∗∗

Handling times follow a power law relationship in all model approaches (Eq. 6b). The capture rates depend on leaf-litter density following an exponential relationship for uncorrected and one-sided densities (Eq. 5a). In the two-sided approach, there is no leaf litter dependence for the capture rate

∗∗∗ *p* < 0.001

∗∗ *p* < 0.01

∗ *p* < 0.05

∙ *p* < 0.1

The consequences of these litter dependencies in capture rates and handling times for the relationship between per capita consumption rates and the amount of leaf litter in the system are illustrated in Fig. 3c–e for three prey densities. While the consumption rates decreased constantly with leaf litter density at low (Fig. 3c, 10 springtails per arena) and intermediate prey densities (Fig. 3d, 100 springtails per arena) under all three spatial corrections, we found a hump-shaped relationship at higher prey densities (Fig. 3e, 600 springtails per arena) for the uncorrected and the one-sided scenario.

## Discussion

In this study, we tested how changing habitat structure in a leaf litter-dominated ecosystem may influence predator–prey interactions by examining functional responses in a laboratory experiment. Contrary to our first hypothesis, we have not found a switch from hyperbolic to sigmoid functional responses with increasingly complex habitat structure. Corroborating our expectations, we found a highly significant decrease in capture rates with increasing litter density except for our analyses correcting for increase in habitat area on both sides of the leaves (two-sided correction) where capture rates remain constant. While we expected handling times to be unaffected by leaf litter density, our analyses revealed decreasing handling times with increasing leaf litter densities.

As the functional responses showed a hyperbolic shape at each litter density, we suppose that the particular habitat structure realised by the beech leaf litter does not provide sufficient hiding refuges for the springtails within the experimental design employed. This may be due to the mobility and the particularly flattened shape of the centipede body, allowing it to explore the interstices between the leaves in a similar fashion to its significantly smaller prey. Subsequent studies need to replicate our experiments for predator groups that differ in their ability to hunt within the interstices between the leaves to address the generality of our result.

Consistent with our initial hypothesis, the capture rates decreased with increasing litter density. As capture rates are composed of encounter rates and attack success (Gergs and Ratte 2009; Vucic-Pestic et al. 2011), we tested whether this effect is caused by (1) a dilution effect reducing encounter rates as increasing litter density yields a higher surface area of the leaves available for interactions, or (2) decreases in the efficiency of the attacks (i.e. capture rates in relation to the available foraging area, or “relative capture rates”) of the centipedes. We found that the significant decrease in capture rates with leaf litter densities is turned into a neutral relationship when accounting for increases in habitat size for springtails and centipedes, as the surface area of the leaves is augmented with an increasing amount of litter. This finding is supported by the observation that centipedes and springtails move on the ground area of the experimental arena as well as on both sides of the leaves. In consequence, our results suggest that the attack efficiency (i.e. the success rate of attacks upon encounters) of the centipedes does not change with litter density, whereas increasing habitat size reduces the encounter rates by diluting the prey population to lower density. The constant capture rates in the analyses correcting for the two-sided increase in habitat size with leaf density show that the dilution effect is responsible for the negative relationship between capture rates and prey density in our experiment.

Beyond that, we found significant decreases in handling time with litter density. We did not anticipate such results and, unfortunately, we can do nothing but conjecture about the biological mechanisms that might be responsible for this finding. As it is well known that centipedes are extremely sensitive to dry conditions (Lithobiids have been shown to prefer 90–100 % relative humidity; Albert 1983), the treatments with higher litter density might have provided more humid conditions. Such more suitable microclimatic conditions for the centipedes might be responsible for the decrease in handling time along the leaf litter density gradient if physiological processes involved in ingestion become more efficient with humidity. Future experiments on habitat structure effects on centipede predation should therefore include better means to control for constant humidity in the arenas or at least measure microclimatic heterogeneity therein. However, other biological processes driven by litter density might also contribute to our results (e.g. centipedes might shirk uncovered areas as a strategy to minimise their own predation risk). Together with earlier studies on habitat structure effects on predation (e.g. Crowder and Cooper 1982; Kaiser 1983; Gotceitas and Colgan 1989; Hoddle 2003; Hohberg and Traunspurger 2005; Hauzy et al. 2010), our findings support the general view that more structural complexity tends to reduce the predators consumption rates. We think it is particularly important to highlight this in the context of food-web modelling approaches as this has two major implications in this field: (1) functional responses that are measured experimentally with the aim to parameterise food-web models should urgently avoid to use oversimplified “Petri-dish” arenas to reveal realistic consumption rates, and (2), our results provide a mechanistic basis to couple the trophic and non-trophic effects of leaf litter in dynamic population models of soil food-webs.

As for any empirical study, some potential caveats need to be mentioned. For example, the functional response models fitted under the spatial corrections did not reach saturation, because correction of the densities compressed the prey density range. This is particularly important for the estimation of handling times in functional response model fitting. However, as our analyses have shown that the general patterns in leaf litter dependency of the functional response parameters also apply for the well-saturated model fittings based on the uncorrected spatial scenario, this should not affect our conclusions. Furthermore, we could have avoided unsaturated curves under the spatial correction scenarios by extending the range in prey densities beyond the maximum of 25,000 individuals per square metre. Besides the experimental impracticability of the extremely high numbers of springtails per treatment, this would also have by far exceeded the densities of natural springtail populations (biomasses of ~0.6 g per m^2^ corresponding to ~4,000 individuals per m^2^; calculations based on dry-weight data from Schaefer 1990 multiplied by water-fraction factor four from Peters 1983). In conclusion, we have decided to keep the springtail densities of our experiment within the range of natural densities while addressing the consequences of natural habitat structures on consumption rates, which avoids the fallacies imposed by oversimplified laboratory conditions (Munyaneza and Obrycki 1997; Vucic-Pestic et al. 2010a).

In soil food webs, springtails are amongst the most abundant taxonomic groups within the microbi-detritivore guild and therefore of critical importance for litter decomposition (Chen and Wise 1997). In a study with a focus on spider predation upon springtails, it has been shown that a reduction of springtails reduces litter decomposition rates (Lawrence and Wise 2000), indicating the importance of top-down regulating mechanisms in soil litter systems. In this study, we present a novel mechanism for how top-down control might be coupled to the dynamics of leaf litter fall with far reaching consequences for decomposition and population dynamics of microbi-detritivores and their predators. The non-trophic effect provided by habitat-altering leaf litter fall can be included in predator–prey functional responses by changing the densities of predators and prey. Hence, future studies dealing with quantitative description of predators and prey in these systems should not only include densities per square metre but additionally provide information on litter densities.

Moreover, the capture rates and handling times are significantly affected by increasing leaf litter densities, but the consequences of these relationships are not straightforward: while decreasing handling times should lead to increasing consumption rates, decreasing capture rates should cause decreasing consumption rates. Our analyses illustrate that consumption rates generally decrease with increasing litter density, except for the combination of the highest springtail density with the lowest litter density. In consequence, the habitat modifications mediated by leaf litter fall and the subsequent decomposition processes might be responsible for regular shifts between bottom-up and top-down control regimes in some leaf litter systems where phases of litter scarcity can occur due to fast decomposition processes (e.g. systems dominated by maple or alder leaf litter) or reduced litter fall. Corresponding patterns in detritivore and predator population dynamics of mixed decidous forests where predator abundances exceed detritivore abundances in the autumn have been documented (Ponsard et al. 2000). However, our results suggest that, in litter-systems with slow decomposition rates (e.g. systems dominated by beech or oak leaf litter), the potential for top-down control of predators on decomposers should be weak. Our findings shed new light on the ongoing debate whether soil litter systems are top-down or bottom-up regulated (de Ruiter et al. 1995; Polis and Strong 1996; Bengtsson et al. 1997; Scheu and Schaefer 1998). Interestingly, they illustrate that non-trophic effects of leaf litter can drive the strength of predatory top-down control. Hence, understanding the importance of top-down and bottom-up control in soil ecosystems requires integrating trophic and non-trophic effects (Fontaine et al. 2011; Kéfi et al. 2012).

## Conclusions

In this study, we have shown how changes in habitat structure affect the predator–prey functional response in leaf-litter systems by diluting predator and prey densities, which reduces their encounter rate. Hence, top-down control of decomposers might be restricted to ecosystems where leaf-litter decomposition is fast enough to deplete habitat structure significantly within one vegetation period. In contrast, many typical temperate forest ecosystems are characterised by slow decomposition rates thus leading to thick litter layers with structured habitats. We have shown that this reduces top-down control by the dilution effect, whereas more complex indirect effect on the efficiency of the attacks could be ruled out. The spatial habitat structure of the litter layer thus determines the strength of predatory top-down pressure, which provides evidence that non-trophic interactions may govern ecosystem organisation.

## Electronic supplementary material

Below is the link to the electronic supplementary material.

Supplementary material 1 (DOC 19.5 kb)
