# Supplementary material for: Habitat structure alters top-down control in litter communities
Source: Oecologia. 2012 Nov 28;172(3):877–87. doi: 10.1007/s00442-012-2530-6 (PMC3679420; doi:10.1007/s00442-012-2530-6)
Supplement: Supplementary file 1 — Supplementary material 1 (DOC 19.5 kb) [file 442_2012_2530_MOESM1_ESM.doc]

**Table S1:** Functional response model evaluation according to their ΔAIC with constant values, power law and exponential relationships in capture rates and handling times, respectively.
